# Radiation reduction in computer-assisted spinal deformity surgery using 3D and 2D pediatric specific low-dose fluoroscopy protocols

**DOI:** 10.1016/j.xnsj.2026.100889

**Published:** 2026-04-12

**Authors:** Jules Cool, Ariena J. Rasker, Jaap M. Groen, Sigrid N.W. Vorrink, Mark C. Altena, Barend J. van Royen, Mario Maas, Thom E. Snijders, Diederik H.R. Kempen

**Affiliations:** a Department of Orthopaedic Surgery, OLVG, Amsterdam, the Netherlands; b Department of Orthopaedic Surgery and Sports Medicine, Amsterdam UMC location University of Amsterdam, Amsterdam, the Netherlands; c Amsterdam Movement Sciences, Musculoskeletal Health - Restoration and Development, Amsterdam, the Netherlands; d Department of Medical Physics, OLVG, Amsterdam, the Netherlands; e Department of Radiology and Nuclear Medicine, Amsterdam UMC location University of Amsterdam, Amsterdam, the Netherlands

**Keywords:** Computer-assisted navigation, Imaging quality, Intraoperative imaging, Low-dose protocols, Pedicle screws, Radiation dose, Spine

## Abstract

**Background:**

Computer-assisted navigation (CAN) facilitates accurate pedicle screw insertion in the surgical correction of adolescent idiopathic scoliosis (AIS), but exposes patients to ionizing radiation via 3D cone-beam CT and 2D fluoroscopy. The aim of this study was to evaluate pediatric-specific low-dose 3D and 2D imaging protocols, assessing their effect on radiation reduction and imaging quality.

**Methods:**

Thirty AIS patients were prospectively enrolled: 10 in the standard 3D protocol and 20 in the low-dose 3D protocol. Standard- and low-dose 2D fluoroscopy was used throughout, independently from 3D dose. Outcome measures included intraoperative radiation dose expressed as dose-area product (DAP), objective image quality (signal-to-noise ratio (SNR) and contrast-to-noise ratio (CNR)), and subjective image quality scoring.

**Results:**

The median 3D radiation dose was significantly lower for the low-dose protocol compared to the standard protocol (24.6 vs. 57.7 cGy*cm^2^, p<.001), without significant differences in SNR or CNR. Both protocols were deemed adequate for pedicle screw navigation in the subjective scoring of image quality. For 2D fluoroscopy, mean total DAP ranged from 18.5 to 106.7 cGy*cm^2^ with the standard protocol and from 15.9 to 61.3 cGy*cm^2^ with the low-dose protocol, depending on frame rate.

**Conclusions:**

The low-dose 3D protocol achieved a radiation reduction of 57%. Despite minor, nonsignificant reductions in SNR and CNR, subjective image quality remained adequate. This supports clinical adoption of the low-dose 3D protocol, offering substantial radiation savings. 2D fluoroscopy also contributed substantially to total intraoperative dose. 2D dose may be reduced by intraoperative measures such as minimizing frame rate.

## Introduction

Pedicle screw placement in adolescent idiopathic scoliosis (AIS) surgery represents a technically demanding procedure due to the presence of dysplastic pedicles and vertebral rotation [1]. Serious complications can occur when pedicle screws are misplaced, leading to a breach in the vertebral cortex and posing risks of damage to structures in the surrounding tissue. These structures include neuronal structures such as the spinal cord or nerve roots, major vessels as the aorta or azygos vein, but also trachea and pleurae [[2], [3], [4]]. In freehand pedicle screw insertion, surgeons rely on anatomical landmarks and 2D imaging (intraoperative fluoroscopy) to guide pedicle screw placement. However, with this technique, pedicle misplacement rates of up to 30% are reported in the literature [4]. Various intraoperative technologies are available that provide superior pedicle screw insertion accuracy compared to freehand pedicle screw insertion. These technologies include advanced imaging (computed tomography (CT)), navigation systems and robot assisted procedures [5].

In AIS surgery, intraoperative cone-beam CT provides 2D fluoroscopy for level confirmation and verification of screw position, and 3D CT-like imaging for pedicle screw navigation. Linked navigation systems subsequently allow for the real-time tracking of surgical instruments and pedicle screws, by superimposing their position onto the intraoperatively acquired 3D image [6]. This technique is also referred to as computer-assisted navigation (CAN), which provides a higher accuracy for screw placement compared to freehand pedicle screw insertion [7]. However, the primary drawback of CAN is the increased intraoperative exposure of patients to ionizing radiation [8,9]. AIS patients are generally of young age and therefore more susceptible to the carcinogenic effects of ionizing radiation compared to adults [10]. It is reported that the use of CAN delivers up to four times the radiation dose compared to solely 2D fluoroscopy [8,11].

To limit the intraoperative radiation exposure in the surgical treatment of AIS, imaging should be conducted with the lowest feasible radiation dosage, according to the As Low As Reasonably Achievable (ALARA) principle [12]. On the other hand, dose reduction causes an increased noise level within the image, potentially compromising imaging quality and affecting the accuracy of pedicle screw navigation [13,14]. The primary aim of this study was therefore to examine the intraoperative radiation exposure and imaging quality of newly developed pediatric specific 3D and 2D low-dose intraoperative imaging protocols, used in the computer-assisted surgical correction of AIS.

## Material and methods

This investigator-initiated single center prospective cohort study was reviewed by the local ethics committee under registration number WO22.145 and approved by the Board of Directors.

### Patient population and inclusion

Thirty AIS patients aged ≤20 years undergoing a thoracic scoliosis correction at our institution (OLVG, Amsterdam, the Netherlands) were included. In all patients, intraoperative CAN was used to guide pedicle screw navigation. Patients treated for other etiologies of scoliosis, as congenital or neuromuscular scoliosis, were excluded. The first 10 patients were treated using a standard 3D fluoroscopy protocol. During this period, a low-dose 3D fluoroscopy protocol was developed which was used in the following 20 patients.

In addition to the low-dose 3D protocol, the 2D fluoroscopy protocol was also adapted to reduce radiation dose. Both standard- and low-dose 2D modes were used throughout this cohort, without a strict per-patient assignment. As the choice of 2D dose level was independent from the 3D dose level within a procedure, the resulting distribution of the 2D datasets could not be directly matched to the 3D dose levels.

### Intraoperative imaging protocols

The Cios Spin mobile C-arm (Siemens Healthineers AG, Erlangen, Germany) was used to acquire intraoperative imaging acquisitions. This iso-centric C-arm features a 30 × 30 cm CMOS (complementary metal oxide semiconductor) flat detector panel and enables for both 2D fluoroscopy and 3D cone-beam CT imaging. The 3D and 2D low-dose protocols were developed by the Research & Development department of Siemens (Siemens Healthineers AG, Forchheim, Germany). Both the standard- and low-dose 3D images were acquired at a tube voltage of 110 kV and variable tube current, with 100 projections recorded in a single 196-degree rotation. A detector entrance dose of 0.087 µGy/image was used for the standard protocol. This dose level was lowered to 0.029 µGy/image for the low-dose protocol. Similar parameters were used for the 3D image reconstructions (0.313 × 0.313 × 0.313 mm^3^ voxel size on a 512 × 512 × 512 volume resolution, 16 × 16 × 16 cm^3^ field of view). To acquire the low-dose 2D fluoroscopy protocol, the per-frame radiation dose was adjusted from 26 nGy to 4 nGy by customized settings for the clinical protocols.

### Surgical procedure

All surgical procedures were performed by two senior spine surgeons trained in freehand pedicle screw placement. CAN had been routinely used at our institution since 2018, prior to the start of this study in 2020. Standard protocols for pediatric spinal deformity surgery were used, including prone patient positioning on a carbon spine table and intraoperative neuromonitoring (motor evoked potentials and somatosensory evoked potential). The desired levels, minus one, were exposed by posterior longitudinal midline incision using anatomical landmarks. A marker was placed at the lowest exposed level and a single posterior-anterior fluoroscopic image was used to identify the exposed level. Subsequently, the exposure was extended cranial and caudal. Since the field of view of the C-arm cannot include all vertebrae planned for instrumentation, the freehand technique was used to place pedicle screws in the two most caudal vertebrae with usually large pedicles. The entry point was made with an awl and metal pins were placed to mark the pedicles. After confirming the correct entry point on a single posterior-anterior fluoroscopic image, the pedicle was probed and the screw was placed.

Subsequently, a reference clamp was attached to the spinous process just outside the field of view and the Cios Spin C-arm was positioned over the surgical field. An intraoperative 3D-run was initiated after the vertebral levels were confirmed using 2D fluoroscopy. Pedicle screw entry points and intrapedicular trajectories were prepared using a navigated awl and probe and pedicle screws were inserted. In cases involving dysplastic or morphologically challenging pedicles, additional posterior-anterior fluoroscopic images were obtained to verify individual screw positions. Subsequently, the scoliosis was corrected using a cantilever technique. The alignment of the spine was checked on posterior-anterior fluoroscopic imaging and additional corrections were made for optimal spinal alignment when needed.

### Radiation exposure

Extracted patient characteristics included age, gender and body mass index (BMI). Collected intraoperative data included the intraoperative imaging protocol used (standard or low-dose 3D and 2D settings), the number of complete vertebrae within the 3D scan, the upper vertebral level as visible on the 3D images and the intraoperative radiation dose administered during the scan. The dose-area product (DAP) noted in cGy*cm^2^ was obtained from the 3D and 2D imaging data to quantify intraoperative radiation exposure. For the 3D imaging, the DAP per projection and total DAP was noted and for the 2D imaging the total number of pulses, radiation time, DAP per frame and total DAP.

### Image quality

Imaging quality of the 3D acquisitions was assessed by measuring the signal-to-noise ratio (SNR), contrast-to-noise ratio (CNR) and surgeon's task-based scoring of imaging quality. The SNR, or image clarity, was calculated by Psignal/Pnoise, where Psignal represented the mean pixel value or true signal level within a region of interest (ROI), and Pnoise the standard deviation of pixel values or fluctuation around the signal level within the same ROI. Regarding ROI placement, the aortic lumen was chosen since it represented a homogeneous region (Figure). In the calculation of the SNR, absolute values were taken to avoid negative SNRs. Contrast-to-noise ratio, or structural distinguishability, was calculated by (Pbone-Psignal)/Pnoise. Pbone was defined as the mean pixel value within a ROI, placed consistently within the same vertebral body across all 3D scans (9th thoracic vertebra). If this vertebra was not included in the 3D scan, or if artifacts compromised image quality at the respective level, the best visible adjacent vertebral body was selected.

**Fig:**
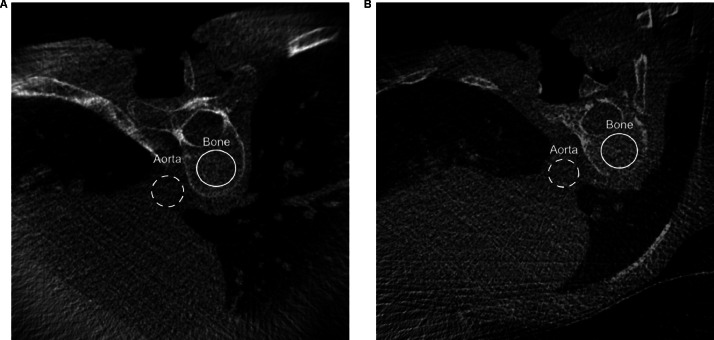
Axial slices from the intraoperative 3D-images using the standard (A) and low-dose (B) protocol, with regions of interest for signal-to-noise ratio and contrast-to-noise ratio calculation illustrated.

All 3D scans were independently scored on a 1 to 5 Likert scale by two spine surgeons (TES and DHRK) and one research physician (JC) after the inclusion period had ended, all of whom were blinded for the protocol used. A score of 1 represented inadequate imaging quality, 2 poor image quality, 3 acceptable image quality, 4 good image quality and 5 excellent image quality (comparable to standard CT of the spine) to safely guide pedicle screw insertion.

### Statistical analysis

IBM SPSS statistics 30 (IBM Corp., Armonk, NY) was used to perform statistical analysis. For continuous variables, data were presented as mean ± standard deviation (SD) with 95% confidence intervals (95% CI), or as median (interquartile range (IQR), range), depending on distribution. If group numbers were considerably low, only mean and range were reported. Hypothesis testing of means was done by Mann-Whitney U test to examine potential significant differences in radiation exposure between the standard- and low-dose 3D fluoroscopy protocols, given the nonparametric distribution of 3D data. The same test was applied to compare patient characteristics across allocation groups.

To compare 2D fluoroscopy outcomes between cohorts while accounting for the frame rates applied within protocols, a linear model was fitted with 2D protocol (standard vs. low-dose) and frame rate (7.5, 10, and 15 frames per second) as fixed factors. For outcomes with a skewed distribution, ln-transformation was applied prior to modeling. Both unadjusted (protocol-only) and frame rate adjusted models were evaluated. Model outputs included p-values and β with 95% CI. To examine significance differences in SNR and CNR between the low-dose and standard 3D protocol, as well as for the subjective score of imaging quality independent samples T-test was used. For the agreement between the three independent assessors of imaging quality, the interclass correlation coefficient (ICC) was determined. An ICC value of >0.90 was considered excellent reliability, 0.75–0.90 good, 0.50–0.75 moderate and <0.50 poor [15]. Statistical significance was defined as p<.05

## Results

### Patient and surgical characteristics

The overall population had a median age of 15.7 years (IQR: 1.9, range 13.7–20.3) years at the time of surgery, with a median BMI of 18.7 kg/m^2^ (IQR: 3.1, range 14.9–28.6) kg/m^2^. A median of 9 levels (IQR: 2, range 5–11) levels were fused to achieve sufficient deformity correction. The majority of the 3D scans were acquired at the thoracic level, with a median of 6 (IQR: 1, range 5–8) vertebrae captured within a single 3D volume. The number of fusion levels was significantly higher for patients in the low-dose protocol group (p=.049).

### Radiation dose

Table 1 shows the results of the radiation dose within each of the 3D imaging protocols. An example of images acquired using the standard- and low-dose protocols is presented in the Figure. In addition, the dose metrics of 2D fluoroscopy for the standard- and low-dose protocols are shown in Table 2. After adjustment for frame rate, no significant between-protocol differences were observed for total pulses (p=.764), 2D radiation time (p=.914), DAP per frame (p=.394) or total DAP (p=.431). Detailed model estimates (unadjusted and frame rate adjusted p-values and β with 95% CI) are provided in the supplementary material (table A.1).

**Table 1:** Patient and surgical characteristics, and 3D imaging dose metrics for the standard- and low-dose 3D protocols.

Imaging protocol				Standard 3D protocol	Low-dose 3D protocol	p-value
Patient characteristics						
	Patients (n)			10	20	
	Males (n)			2	8	
	Age (years), median (IQR, range)			15.7 (1.7, 14.0–19.1)	15.7 (2.1, 13.7–20.3)	.948
	BMI (kg/m^2^), median (IQR, range)			18.6 (3.3, 16.9–21.5)	18.9 (4.1, 14.9–28.6)	.502
Surgical characteristics						
	Fusion levels (n), median (IQR, range)			8 (4, 5–11)	9 (1, 8–11)	.049
	Vertebra within 3D volume (n), median (IQR, range)			6 (2, 5–7)	6 (1, 5–8)	.307
	Upper vertebral level in 3D scan					
			T3	-	2	
			T4	2	8	
			T5	2	4	
			T6	1	6	
			T7	-	-	
			T8	-	-	
			T9	3	-	
			T10	1	-	
			T11	1	-	
Radiation exposure from intraoperative 3D imaging						
		Detector entrance dose (µGy/projection)		0.087	0.029	
		Projections in a 3D rotation (n)		100	100	
		DAP 3D per projection (cGy*cm^2^), median (IQR, range)		0.55 (0.29, 0.39–0.84)	0.25 (0.13, 0.15–0.72)	<.001
		Total DAP 3D (cGy*cm^2^), median (IQR, range)		57.7 (28.2, 46.8–84.2)	24.6 (12.9, 15.3–71.8)	<.001

BMI, body mass index; DAP, dose-area product; IQR, interquartile range.

**Table 2:** Dose metrics for the standard- and low-dose 2D protocols.

Imaging protocol	Standard 2D protocol			Low-dose 2D protocol			Adjusted p-value*
Imaging settings							
Frames per second (n)	7.5	10	15	7.5	10	15	
Per-frame radiation dose (nGy)	26	26	26	4	4	4	
Outcomes†							
Patients (n)	3	0	2	2	6	15	
Total pulses (n), mean (range)	77.5 (54–101)	-	259.7 (169–321)	95.1 (27–204)	160.8 (82–285)	207.0 (169–245)	.764
2D radiation time (sec), mean (range)	15.5 (10.9–20.1)	-	26.1 (16.8–31.2)	19.2 (7.4–34.9)	24.3 (11.9–37.6)	22.1 (21.2–22.9)	.914
DAP/frame (cGy*cm^2^), mean (range)	0.3 (0.1–0.6)	-	0.4 (0.4–0.5)	0.2 (0.0–0.4)	0.5 (0.1–1.0)	0.3 (0.2–0.3)	.394
Total DAP (cGy*cm^2^), mean (range)	18.5 (4.6–32.4)	-	106.7 (75.8–128.2)	15.9 (2.0–69.7)	61.3 (25.1–111.4)	58.3 (38.3–78.4)	.431

DAP, dose-area product; SD, standard deviation.

⁎ Adjusted p-values from a linear model with protocol and frames per second included as fixed factor.

† Total DAP is influenced by several interrelated factors beyond the per-frame radiation dose selected in the low-dose or standard 2D fluoroscopy protocol. This table reports 2D fluoroscopy dose metrics across different imaging parameters. For each protocol, input settings (per-frame radiation dose (nGy) and frame rate (frames per second), exposure characteristics (total number of pulses and fluoroscopy time), and dose outputs (DAP per frame and total DAP) are presented.

### Imaging quality of 3D volumes

For the standard and low-dose 3D volumes, the SNR was 0.6±0.4 (range 0.0–1.2) and 0.5±0.3 (range 0.0–1.2), respectively. The corresponding CNR values were 1.6±0.6 (range 0.9–2.6) for the standard-dose protocol and 1.3±0.4 (0.6–2.4) for the low-dose protocol (Table 3). No statistical difference in SNR or CNR were observed between the standard and low-dose 3D protocols. For the purpose of pedicle screw navigation, all assessors rated the imaging volumes minimally as acceptable (Likert ≥3), with most ratings being good or excellent (Likert 4–5) (Table 4). Interobserver agreement was low, with an ICC=0.34 (95% CI: −0.21 to 0.66).

**Table 3:** Objective and subjective imaging quality metrics for standard- and low-dose 3D protocols.

	Standard 3D protocol	Low-dose 3D protocol	p-value
SNR, mean ± SD (95% CI)	0.6±0.4 (0.0–1.2)	0.5±0.3 (0.0–1.2)	.640
CNR, mean ± SD (95% CI)	1.6±0.6 (0.9–2.6)	1.3±0.4 (0.6–2.4)	.059
Subjective imaging quality (5 point Likert scale), mean ± SD (95% CI)	4.1±0.3 (3.9–4.3)	4.1±0.2 (4.0–4.2)	.465

95% CI, 95% confidence interval; CNR, contrast-to-noise-ratio; SD, standard deviation; SNR, signal-to-noise ratio.

**Table 4:** Frequency distribution of subjective image quality ratings (Likert 1–5) across observers (n=30 scans).

Likert score	Observer 1, n (%)	Observer 2, n (%)	Observer 3, n (%)
1 (inadequate)	0 (0%)	0 (0%)	0 (0%)
2 (poor)	0 (0%)	0 (0%)	0 (0%)
3 (acceptable)	1 (3%)	1 (3%)	0 (0%)
4 (good)	27 (90%)	27 (90%)	24 (80%)
5 (excellent)	2 (7%)	2 (7%)	6 (20%)

## Discussion

The current study showed a substantial reduction in intraoperative radiation dose during 3D imaging acquisitions for pedicle screw navigation in the treatment of AIS by using a pediatric-specific low-dose 3D imaging protocol. Overall, reducing the entrance dose level resulted in a 57% decrease in radiation dose. Although the detector target dose was reduced by threefold (0.029 vs. 0.087 μGy/imaget), the observed twofold reduction in total DAP is consistent with the exponential X-ray absorption law, whereby a threefold change at the detector typically results in approximately a twofold change at the patient level [16]. Despite this reduction in radiation dose during the 3D acquisition, the imaging quality with the low-dose protocol remained adequate for pedicle screw navigation. Objective image quality metrics showed comparable SNR values and slightly lower CNR values, which were not statistically significant. In addition, both 3D protocols provided sufficient imaging quality for pedicle screw navigation in the subjective scoring of imaging quality. Although the interclass correlation coefficient was low (ICC=0.34), this likely reflects the limited variability in scores, as nearly all scans were consistently rated as good or excellent by all observers. These findings support the clinical implementation of pediatric low-dose 3D imaging protocols in AIS surgery, enabling radiation dose reduction while maintaining adequate imaging quality for pedicle screw navigation.

Overall, comparison with previous literature is challenging, as many studies report dose-length product (eg, with the frequently used Medtronic O-arm), whereas the Cios Spin 3D C-arm is a cone-beam system for which the dose-area product (DAP) is applicable. However, a previous cadaveric study by Foster et al. [17] analyzed the 3D radiation dose emitted by the Cios Spin C-arm, and reported DAP for three dose levels (low, medium and high-dose). This study reported a minimum DAP of 645.68 cGy*cm^2^ for the low-dose protocol, and a maximum DAP of 1593.78 cGy*cm^2^ for the high-dose protocol. The average DAP in the current study was substantially lower, for both the standard as well as the low-dose protocol (57.74 cGy*cm^2^ and 24.61 cGy*cm^2^ respectively). This difference is likely attributable to the lowest number of projections per 3D rotation (n=100) in both protocols, combined with a further reduction in entrance dose level for the low-dose protocol. Furthermore, the average BMI was relatively low, which strongly influences radiation dose in (cone-beam) CT imaging [18,19]. These findings suggest that lowering the number of projections, in combination with adjustment of the entrance dose level, is an effective strategy for reducing radiation exposure during intraoperative 3D imaging.

Several limitations of current study must be acknowledged. First, technical phantom testing showed that it was technically feasible to lower the radiation dose further than used in current low-dose 3D protocol. Nevertheless, a safe margin in radiation dose was applied in the actual surgical procedures, to prevent the need for an additional 3D-run due to insufficient imaging quality, or the risk of pedicle screw malpositioning. Secondly, since a postoperative CT is not part of routine clinical care, it remains unknown whether a difference in screw position exists between the standard and low-dose 3D protocols. However, both modalities provided imaging of sufficient quality for pedicle screw navigation, as indicated by the surgeons' subjective assessment of image quality. Furthermore, to ensure a reliable comparison of the radiation dose and imaging quality between the 3D imaging protocols, the inclusion criteria mandated the need for 100 projections in a single 3D-run. However, factors like patients' body habitus might necessitate an increased number of projections to enhance imaging quality. Nonetheless, since the radiation dose per projection was lowered in the low-dose protocol, the same effect in radiation reduction can reasonably be expected for scans that are acquired using a larger number of projections. The study also used a non-randomized sequential design, which may introduce temporal confounding. However, the Cios Spin system and navigation workflow had been routinely used before the initiation of this study, making a substantial learning-curve effect during the study unlikely. Nevertheless, residual confounding related to the study design cannot be entirely ruled out. Finally, the nonuniform allocation of standard- and low-dose 2D fluoroscopy protocols across patients represents a methodological limitation that restricts the interpretability of the between-protocol comparison, and results should be interpreted with caution.

### Clinical implications

In pediatric spinal deformity surgery, surgeon should focus on methods that allow for accurate pedicle screw insertion, without an excessive amount of radiation. While the use of computer-assisted navigation shows significantly safer pedicle screw insertion compared to the freehand or 2D-fluoroscopy guided technique, the intraoperatively acquired (cone-beam) CT-scan generally involves considerable radiation exposure [8,20]. Using aforementioned strategies, the current study shows that this intraoperative radiation exposure can be reduced substantially, without significantly compromising imaging quality. However, while no statistically significant differences were observed in SNR and CNR, CNR was generally slightly lower with the low-dose settings. Nevertheless, this did not hinder the intraoperative delineation of critical anatomical structures such as pedicles. Thus, surgeons may be willing to accept a slight reduction in imaging quality if the acquired images still allow for safe pedicle screw navigation, in exchange for a substantial reduction in radiation exposure.

Due to heterogeneity in 2D fluoroscopy settings, which complicates direct between-protocol comparisons, adjusted analyses for frame rate were performed. These analyses did not show clear between-protocol differences in 2D metric, which should be interpreted cautiously given the low numbers included in the analysis. Nevertheless, 2D fluoroscopy contributed substantially to the overall intraoperative dose. In some cases, the total 2D fluoroscopy DAP matched the 3D acquisition DAP, even though radiation-safety efforts usually focus on 3D imaging. The variability in 2D dose across patients is likely attributable to differences in frames-per-second settings and additional fluoroscopic verification of individual screw positions in cases with challenging pedicle anatomy. Besides the per-frame radiation dose as a measure to potentially lower radiation exposure, additional factors should be considered intraoperatively. It should also be noted that the total DAP is determined not only by technical parameters but also greatly influenced by patient-related and geometric factors, such as BMI and viewing angle of the C-arm.

To reduce intraoperative 2D dose, first of all, the frame rate of the C-arm can be adjusted by the radiographer on the console and reduced as needed to limit exposure, e.g., from 15 to 7.5 frames per second. Therefore, we recommend surgeons to discuss target frame rate and dose settings together with the radiographer just before surgery to ensure awareness and minimize exposure. Secondly, dose accumulates when the foot pedal or hand switch is pressed. As determined by current regulation (IEC 60601-2-54:2022, cl. 203.6.2.1. Normal initiation and termination of the IRRADIATION), it is not possible for the manufacturer to replace this user-controlled activation with a fixed per image exposure time, despite such limit would likely reduce total dose. Long continuous fluoroscopy should therefore be avoided, with short activations used instead. Most importantly, surgeons should be aware that 2D radiation dose can reach considerable dose levels, for both the patient and surgical personnel, and easy adjustments as described above can lower the radiation dose. The influence of adjustments to frame rate settings and manual exposure time on 2D radiation dose were not taken into consideration in this study since the surgeons became aware of these effects during the analysis of the results after inclusion had ended.

## Conclusion

Using a pediatric-specific 3D cone-beam imaging protocol, a 57% reduction in radiation exposure was achieved during the computer-assisted surgical correction of AIS. At the same time, neither subjective nor objective imaging quality differed significantly between the standard and low-dose 3D protocols. As a result, this adjusted pediatric specific imaging protocol closely adheres to the ALARA principle, as treatment quality is preserved while radiation exposure is minimized. In addition, 2D fluoroscopy was found to contribute considerably to total radiation dose. Besides reducing per-frame radiation dose, user-dependent factors such as optimization of frame rate and fluoroscopy activation time may provide further opportunities for dose reduction.

## Author contributions

Jules Cool: Formal analysis, Investigation, Data Curation, Writing—Original Draft. Ariena J. Rasker: Conceptualization, Methodology, Investigation, Project administration, Writing—Review and Editing, Supervision. Jaap Groen: Conceptualization, Resources, Writing—Review and Editing, Supervision. Sigrid Vorrink: Conceptualization, Methodology, Writing—Review and Editing, Supervision, Project administration. Mark Altena: Conceptualization, Writing—Review and Editing, Supervision. Barend J. van Royen: Investigation, Writing—Review and Editing, Supervision. Mario Maas: Validation, Writing—Review and Editing, Supervision. Thom E Snijders: Investigation, Writing—Review and Editing, Supervision. Diederik H.R. Kempen: Conceptualization, Methodology, Investigation, Writing—Review and Editing, Supervision, Project administration

## Funding

The research was supported by an academic-industry collaboration with Siemens Healthineers (Forcheim, Germany).

## Declarations of competing interests

The authors declare that they have no known competing financial interests or personal relationships that could have appeared to influence the work reported in this paper.

## Appendix Supplementary materials

Image, application 1
