# Supplementary material for: Radiation reduction in computer-assisted spinal deformity surgery using 3D and 2D pediatric specific low-dose fluoroscopy protocols
Source: N Am Spine Soc J. 2026 Apr 12;26:100889. doi: 10.1016/j.xnsj.2026.100889 (PMC13196465; doi:10.1016/j.xnsj.2026.100889)
Supplement: Supplementary file 1 [file mmc1.docx]

| **Outcome** | **Unadjusted β (95% CI)**^a^ | **p-value** | **Adjusted β (95% CI)**^a^ | **p-value** |
| --- | --- | --- | --- | --- |
| Total pulses (n) | 64.800 (-15.08 - 144.68) | 0.107 | 10.821 (-62.84 - 84.49) | 0.764 |
| 2D radiation time (sec) | 1.101 (-7.86 - 10.06) | 0.803 | -0.553 (-11.00 - 9.89) | 0.914 |
| DAP/frame^b^ (cGy*cm^2^) | 0.60 (-0.41 - 1.60) | 0.232 | 0.441 (-0.61 - 1.49) | 0.394 |
| Total DAP^b^ (cGy*cm^2^) | 0.98 (-0.32 - 2.27) | 0.133 | 0.435 (-0.69 - 1.56) | 0.431 |

**Table A.1:** Protocol effect of standard dose compared to low-dose on 2D fluoroscopy outcomes (n=28; for ln-transformed outcomes, β is reported on the ln scale)

Abbreviations: 95% CI, 95% confidence interval; DAP, dose-area product

^a^Unadjusted model included protocol only; adjusted model included protocol and frame rate (7.5/10/15 frames per second) as fixed factors

^b^ln transformed; β reported on the ln scale
